# Immunogenicity and Determinants of Antibody Response to the BNT162b2 mRNA Vaccine: A Longitudinal Study in a Cohort of People Living with HIV

**DOI:** 10.3390/vaccines12101172

**Published:** 2024-10-16

**Authors:** Tatjana Baldovin, Davide Leoni, Ruggero Geppini, Andrea Miatton, Irene Amoruso, Marco Fonzo, Chiara Bertoncello, Mascia Finco, Maria Mazzitelli, Lolita Sasset, Annamaria Cattelan, Vincenzo Baldo

**Affiliations:** 1 Department of Cardiac, Thoracic, Vascular Sciences and Public Health, University of Padua, 35131 Padua, Italyruggero.geppini@studenti.unipd.it (R.G.); andrea.miatton@studenti.unipd.it (A.M.); irene.amoruso@unipd.it (I.A.); marco.fonzo@unipd.it (M.F.); vincenzo.baldo@unipd.it (V.B.); 2 Infectious and Tropical Diseases Unit, Padua University Hospital, 35131 Padua, Italy; davide.leoni@aopd.veneto.it (D.L.); mascia.finco@studenti.unipd.it (M.F.); lolita.sasset@aopd.veneto.it (L.S.); annamaria.cattelan@aopd.veneto.it (A.C.); 3 Department of Molecular Medicine, University of Padua, 35131 Padua, Italy

**Keywords:** COVID-19, SARS-CoV-2, vaccination, PLWH, HIV, immunogenicity, antibody persistence, booster dose

## Abstract

Background: The COVID-19 pandemic posed significant challenges worldwide, with SARS-CoV-2 vaccines critical in reducing morbidity and mortality. This study evaluates the immunogenicity and antibody persistence of the BNT162b2 vaccine in people living with HIV (PLWH). Methods: We monitored anti-SARS-CoV-2 Spike IgG concentration in a cohort of PLWH at five time points (T0–T4) using chemiluminescent microparticle immunoassays (CMIAs) at the baselined both during and after vaccination. In severely immunocompromised individuals, a boosting dose was recommended, and participants and IgG concentration were measured in the two subgroups (boosted and not boosted). Results: In total, 165 PLWH were included, and 83% were male with a median age of 55 years (IQR: 47–62). At T1, 161 participants (97.6%) showed seroconversion with a median of IgG values of 468.8 AU/mL (IQR: 200.4–774.3 AU/mL). By T2, all subjects maintained a positive result, with the median anti-SARS-CoV-2 Spike IgG concentration increasing to 6191.6 AU/mL (IQR: 3666.7–10,800.8 AU/mL). At T3, all participants kept their antibody levels above the positivity threshold with a median of 1694.3 AU/mL (IQR: 926.3–2966.4 AU/mL). At T4, those without a booster dose exhibited a marked decrease to a median of 649.1 AU/mL (IQR: 425.5–1299.8 AU/mL), whereas those with a booster experienced a significant increase to a median of 13,105.2 AU/mL (IQR: 9187.5–18,552.1 AU/mL). The immune response was negatively influenced by the presence of dyslipidaemia at T1 (aOR 4.75, 95% CI: 1.39–16.20) and diabetes at T3 (aOR 7.11, 95% CI: 1.10–46.1), while the use of protease inhibitors (aORs 0.06, 95% CI: 0.01–0.91) and being female (aOR 0.02, 95% CI: 0.01–0.32) at T3 were protective factors. Conclusions: The immunogenicity of the BNT162b2 vaccine in PLWH has been confirmed, with booster doses necessary to maintain high levels of anti-SARS-CoV-2 Spike IgG antibodies, especially in patients with comorbidities. These findings underline the importance of a personalized vaccination strategy in this population.

## 1. Introduction

The COVID-19 pandemic emerged as a severe threat to global health and economic development [1]. In October 2024, there have been more than 776 million confirmed COVID-19 cases worldwide, including almost 7.07 million deaths and over 13.64 billion administered vaccine doses [2]. Mortality and morbidity associated with COVID-19, together with a large-scale economic burden, promoted an incredibly rapid development and approval of dedicated vaccines, such as novel nucleic acid-based vaccines (e.g., Pfizer-BioNTech/BNT162b2), non-replicating viral vector vaccines (e.g., Janssen/JAd26.COV2.S) and traditional inactivated whole virus vaccines (e.g., Sinovac Biotech/CoronaVac) [3,4]. The safety, immunogenicity, and effectiveness of these vaccines have been evaluated by clinical trials. Nevertheless, vaccine trials and population studies have included a limited number of immunocompromised individuals [5,6,7]. Some recent reviews discussed this topic in detail [8,9,10,11,12,13,14]. Immunocompromised patients (3% of the adult population) could present immunity suppression or over-activation due to their primary health condition and/or concurrent therapy. Additional data are still needed for immunocompromised patients as the COVID-19 spectrum of disease and viral spreading appear more prolonged and severe [10].

According to the WHO report, HIV is an independent risk factor for COVID-19 mortality [15]. PLWH, particularly those with high comorbidity burdens, uncontrolled viral loads, or low CD4+ T cell counts, are more susceptible to severe COVID-19 outcomes [16,17]. This vulnerability has warranted enhanced health care oversights since the pandemic’s onset [18,19]. Specifically, PLWH not on antiretroviral therapy, with detectable viral loads and low CD4+ T cell counts (<200 cells/mm^3^) and/or with low CD4+ T cell count nadir, showed a higher risk of developing an anomalous immune response—characterized by delayed or insufficient antibody production after SARS-CoV-2 infection, as well as a greater risk of severe COVID-19 [20,21,22,23]. During the COVID-19 pandemic, the early and widespread distribution of anti-SARS-CoV-2 vaccines showed its paramount importance in reducing COVID-19 mortality among the general population [24]. Investigations on the efficacy of new vaccinations among subpopulations with an impaired immunity response are frequently poor in the early phases of vaccines development and registration trials [25]. Considering the more severe impact of infections on immunosuppressed individuals, vaccination is a first-line strategy for their protection; therefore, vaccine immunogenicity evaluations among these subgroups are a matter of utmost priority.

Immunization responses in PLWH have shown varying vaccine efficacy historically. Factors such as CD4+ T cell count, uncontrolled viral load, and HIV disease stage closely correlate with lower protective antibody responses post-vaccination [26,27]. Studies indicate that PLWH on combination antiretroviral therapy (cART) often exhibit suboptimal serologic responses to vaccines like hepatitis B or influenza when compared to the general population [28,29,30,31]. However, evidence suggests that the SARS-CoV-2 vaccination in PLWH is safe and effective in preventing severe infection, with antibody responses similar to the general population [7,32,33]. Ongoing research is crucial to determine the persistence of vaccine-induced protection and anti-SARS-CoV-2 Spike IgG concentration (hereafter referred to as anti-Spike IgG) in PLWH, with recent data indicating a strong correlation between neutralizing antibodies, targeting anti-Spike IgG, and overall protection from SARS-CoV-2 infection [32,34]. Long-term immunological data involving both humoral and cellular response is needed to confirm these findings, even though studies on T cellular responses to these vaccines are technically difficult, and data are still scarce [32,35].

In some studies, PLWH with a CD4+ T cell count < 200/mm^3^, detectable viral loads, and an advanced age seem to be unable to mount a robust humoral response after SARS-CoV-2 vaccination, demonstrating reduced rates of seroconversion and neutralization even after receiving a booster dose [36]. However, according to a systematic review of 34 studies, conversion rates of PLWH for the first and second doses were 67.51% and 96.65%, respectively, showing no difference from controls without HIV [37]. Interestingly, several studies have shown how cART-induced CD4+ T cell recovery might enhance vaccination immunogenicity [38,39].

Our main objective was to determine whether vaccine-derived SARS-CoV-2 anti-Spike IgG concentrations persist in a cohort of PLWH who received the BNT162b2 vaccine in a long-term follow-up (over 6 months) after the completion of the primary vaccination cycle of two doses. The secondary objective of the study was to investigate the impact of immunovirological variables, demographic characteristics, and comorbidities on the production and durability of anti-Spike IgG levels in this patient population.

## 2. Materials and Methods

### 2.1. Study Design

A cohort of PLWH attending the University Teaching Hospital’s Infectious and Tropical Diseases Unit of Padua, Italy, was enrolled in a longitudinal study to evaluate the serological kinetics of vaccine-derived SARS-CoV-2 anti-Spike (anti-S) IgG. At enrolment, demographic and clinical data were collected from the patient’s medical records, including information on previous COVID-19 diagnoses. The study was conducted in accordance with relevant guidelines and regulations, respecting the privacy of patients as approved by the Ethics Committee of the Padua University Hospital (study protocol 147/AO/2021, 13 July 2021). Informed written consent was obtained from all the participants included in the study.

Individuals who had previously been diagnosed with COVID-19, specifically people who tested positive on a molecular swab or had a positive serological test for anti-nucleocapsid (anti-N) antibodies, were excluded from the study.

Enrolled participants were assessed at five distinct time points over a 7-month follow-up period. All recruited PLWHs received stable care from the mentioned Operative Unit. They were regularly monitored for parameters related to HIV progression, e.g., CD4 count, CD4/CD8 ratio, and HIV viral load. Clinical, laboratory, and demographic data were retrieved from medical records. Following enrolment, all participants received the Pfizer-BioNTech BNT162b2 mRNA vaccine in early 2021.

The time point before the first vaccination dose was designated as T0 (the day of the first doses, just before the administration), aiming to assess anti-S IgG antibody levels at the baseline. The same parameters were then assessed at T1 (post-first dose) and 21 days after the first vaccination, during the same session as the administration of the second dose [38,40]. The T2 assessment (short-term) was conducted 1 month after the completion of the 2-doses and full vaccination cycle. T3 (medium-term) and T4 (long-term) were set at 3 months and 6 months after the completion of the primary vaccination cycle (two doses), respectively. At each time point, HIV-related parameters were registered, and the anti-Spike IgG concentration was assessed. Anti-N IgG serology was performed only at T0 and T4 to detect the possible occurrence of natural SARS-CoV-2 infection.

**Figure 1:**
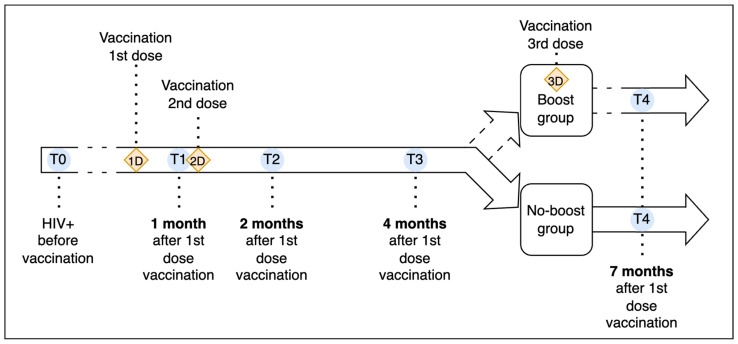
Study timeline indicating the timing of assessments and vaccinations.

In September 2021, the Italian Medicines Agency (AIFA) advised administering a third dose (booster) following the primary vaccination cycle for severely immunocompromised individuals, including those living with HIV (PLWH). This recommendation led to the administration of an early booster dose (i.e., between T3 and T4, approximately 4 months after the second dose) [41,42]. This allowed the dichotomization of our study path, dividing the original cohort into two sub-groups: subjects with the booster dose (boost group) and subjects without the booster (no-boost group). The chronological progression of this study is visually depicted in Figure 1.

T0 represents the baseline evaluations of participants before any vaccination. The first dose was administered shortly after T0. T1 (1 month after the first dose) is the follow-up evaluation, during which the second dose was administered. T2 (short-term, 2 months post-first dose), T3 (mid-term, 4 months post-first dose), and T4 (long-term, 7 months post-first dose) mark subsequent assessments. At T4, participants were split into a boost group, which received a third dose between T3 and T4, and a no-boost group, which did not receive any booster.

### 2.2. Laboratory Methods

Blood samples were collected in BD Vacutainer^TM^ Serum tubes from each participant at every study time point. Serum was separated from whole blood within 4 h from collection by centrifugation (10 min at 1400× *g*) and tested within 24 h. Two automated chemiluminescent microparticle immunoassays (CMIAs) were used to assess antibody levels. The SARS-CoV-2 IgG assay (Abbot Laboratories, Chicago, IL, USA) was used for the qualitative detection of anti-N IgG antibodies to the nucleocapsid protein and the AdviseDx SARS-CoV-2 IgG II (Abbot Laboratories, Chicago, IL, USA) was used for the quantitative determination of anti-S IgG antibodies to the receptor binding domain (RBD) of the spike protein of SARS-CoV-2. Consistently, anti-S antibodies were tested at every time point, whereas the anti-N IgG was tested only at T0 and T4 to monitor the background anti-S IgG antibody levels at the baseline of recruited subjects. The Anti-N IgG concentration was considered positive with chemiluminescent ≥ 1.40, as per the manufacturer’s recommendations. Similarly, the positive cut-off for anti-S IgG was set at 50 AU/mL [43].

### 2.3. Data Analysis

Minimum value, the 25th percentile, median, 75th percentile, and maximum value for anti-Spike IgG concentrations, CD4+ T cell count, CD4/CD8 ratio, and HIV-RNA at different follow-up time points were calculated, and results were used to build box-plot graphs. To longitudinally analyze anti-S IgG antibody levels between different time points, the Friedman test with Dunn’s post-test was used, with separate tests for the two subgroups at T4. *P*-values were reported. Linear regression analysis was performed to evaluate a potential relationship between the anti-Spike IgG concentration and CD4+ T cell count, CD4/CD8 ratio, and HIV-RNA. Any discrepancies between the so-called ‘boosted group’ and the ‘non-boosted group’ for all studied dichotomous and continuous variables were evaluated using Fisher’s exact test and the Mann–Whitney test. Two-sided *p*-values were reported. To identify factors potentially associated with a lower anti-Spike IgG concentration at different time points, logistic regression analysis was performed at each time point, setting as the outcome (the dependent variable) the probability of being in the lowest quartile for anti-Spike IgG concentration. All other investigated variables, sex, BMI, ART regimen (i.e., integrase strand transfer inhibitor (INI), protease inhibitor (PI), non-nucleoside reverse transcriptase inhibitor (NNRTI), and dual or triple therapy) and comorbidities (i.e., dyslipidemia, cardiovascular disease, depression, liver disease, lung disease, renal insufficiency/dialysis, diabetes, solid tumors, hematologic neoplasms, autoimmune diseases, hepatitis B and C, and syphilis, the occurrence of AIDS-related event, CD4+ nadir, CD4+ count, CD4/CD8 ratio, and HIV-RNA) were entered as potential predictors (independent variables). Unadjusted (ORs) and adjusted odds ratios (aORs) and relative 95% confidence intervals were reported. IBM SPSS Statistics 29.0.1.0 was used to perform statistical analysis.

## 3. Results

### 3.1. Study Population

For the present study, 180 PLWH were recruited, but 15 were excluded due to previous SARS-CoV-2 infection. Therefore, 165 PLWH were included and completed the study follow-up; 137 (83%) were males, with a median age of 55 years (IQR: 47–62 years). The median CD4+ T cell count was 687 cells/µL, and the median CD4+ T cell count at nadir was 270 cells/µL; overall, 127 (77%) individuals had CD4+ T cell counts > 500 cells/µL and 38 (23%) had CD4+ T cell counts ranging from 200 to 500 cells/µL. In 161 subjects (97.6%), the HIV-RNA was <50 copies/mL. Regarding the stages of HIV infection, 42 subjects (25.8%) had a previous AIDS event in their medical history. Among the recruited PLWHs, 35 (21.2%) initiated combination antiretroviral therapy (cART) within one year of their HIV diagnosis, while 130 (78.8%) started their treatment more than one year after being diagnosed. The sociodemographic characteristics and comorbidities of the study population are detailed in Table 1.

### 3.2. Immunogenicity and Antibody Response

**Figure 2:**
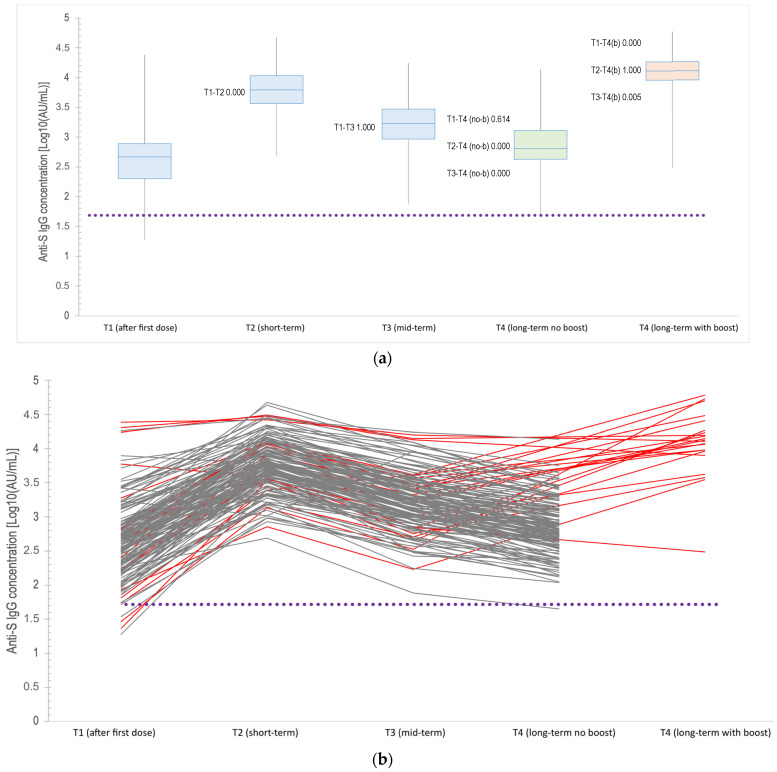
(**a**). Antibody concentrations of anti-SARS-CoV-2 Spike IgG throughout the follow-up time point (T1 to T4). Friedman test with Dunn’s post test (the *p*-value is reported for each pair in the figure). The boxplot shows the concentrations of anti-SARS-CoV-2 Spike IgG at five follow-up time points (T1 to T4), measured in Arbitrary Units per milliliter (AU/mL). The x-axis represents the time points, while the y-axis is on a logarithmic scale, indicating IgG concentration levels. T1 (after first dose): The antibody concentrations range from a minimum of 18.9 AU/mL to a maximum of 24,210.8 AU/mL, with a median of 468.8 AU/mL. The interquartile range (IQR) spans from 200.4 to 774.3 AU/mL. T2 (short-term): Concentrations range from a minimum of 487.7 AU/mL to a maximum of 47,441.1 AU/mL, with a median of 6191.6 AU/mL. The IQR is from 3666.7 to 10,800.8 AU/mL, showing a notable increase after the second dose. T3 (mid-term): The minimum concentration is 76.5 AU/mL, the maximum is 17,367.6 AU/mL, and the median is 1694.3 AU/mL. The IQR extends from 926.3 to 2966.4 AU/mL, showing a reduction compared to T2, but this is still above baseline. T4 (long-term, no boost): For participants who did not receive a booster, concentrations range from a minimum of 44.9 AU/mL to a maximum of 13,804.9 AU/mL, with a median of 649.1 AU/mL. The IQR is from 425.5 to 1299.8 AU/mL, indicating a significant decline in antibody levels. T4 (long-term, with boost): For participants who received a booster dose, concentrations range from 307.3 AU/mL to a maximum of 60,515.1 AU/mL. The median is 13,105.2 AU/mL, and the IQR ranges from 9187.5 to 18,552.1 AU/mL, demonstrating a substantial increase in antibody levels post-booster. The dotted line represents the positivity threshold of 50 AU/mL for the anti-SARS-CoV-2 Spike IgG concentration. (**b**). Spaghetti plot illustrating the anti-SARS-CoV-2 Spike IgG antibody concentrations (×10³ AU/mL) across the follow-up time points (T1 to T4) on a log10 scale. Each line represents an individual patient’s trajectory, with red lines indicating patients who received a booster dose. A dashed line indicates a positive cut-off at 50 AU/mL. This plot highlights the dynamic changes in antibody levels over time, comparing boosted and non-boosted groups, and facilitates better visualization through the use of log10-transformed data.

Figure 2 depicts the vaccine-derived anti-S IgG concentration at each time point throughout the follow-up period. At T0, all subjects had negative serology for both SARS-CoV-2 anti-N and anti-S IgG. At T1 (1 month after the first dose), 161 (97.6%) participants tested anti-S IgG-positive (>50 AU/mL), with a median level of 468.8 AU/mL (IQR: 200.4–774.3 AU/mL). Only four subjects did not show a response to the first dose. At T2 (1 month after the second dose), all subjects maintained anti-S IgG positivity, with an increase in the median antibody concentrations (median 6191.6 AU/mL, IQR: 3666.7–10,800.8 AU/mL). At this time point, people who were seronegative at T1 developed an anti-S IgG concentration (with a median of 1935, IQR: 960–3370 AU/mL). At T3, all the subjects maintained an antibody concentration above the positivity threshold, with a median of 1694.3 AU/mL and IQR of 926.3–2966.4 AU/mL. The distribution of anti-S IgG antibody levels at different time points was not the same for all the pairs (*p* < 0.005), except for T1–T3 (*p* = 1.000); T1–T4 (no boosted group) (*p* = 0.614); and T2–T4 (boosted group) (*p* = 1.000).

### 3.3. Impact of Booster Dose

According to the administration of a booster dose, between T3 and T4 (after the completion of the primary vaccination cycle), our cohort was further divided into two sub-groups (boosted, *n* = 22, 13.3%, and not boosted = 143, 86.7%). The two subgroups were homogeneous in all the characteristics studied (e.g., demographics, medical history, HIV-related parameters, *p* > 0.05), except for sex (males in the no boost group were 80.4%, in the boost group 100.0%, *p* = 0.03) and for the presence of hematological malignancies (Supplementary Table S1). Subjects who did not receive a booster dose exhibited a marked decline in antibody levels, with an IQR ranging from 425.5 to 1299.8 AU/mL and median of 649.1 AU/mL. In contrast, those who received a booster dose showed a considerable increase in anti-Spike IgG concentrations, with an IQR ranging from 9187.5 to 18,552.1 AU/mL and median of 13,105.2 AU/mL and a significant rise in the extremes (Figure 2a,b).

### 3.4. CD4+ Count and HIV Viral Load

**Figure 3:**
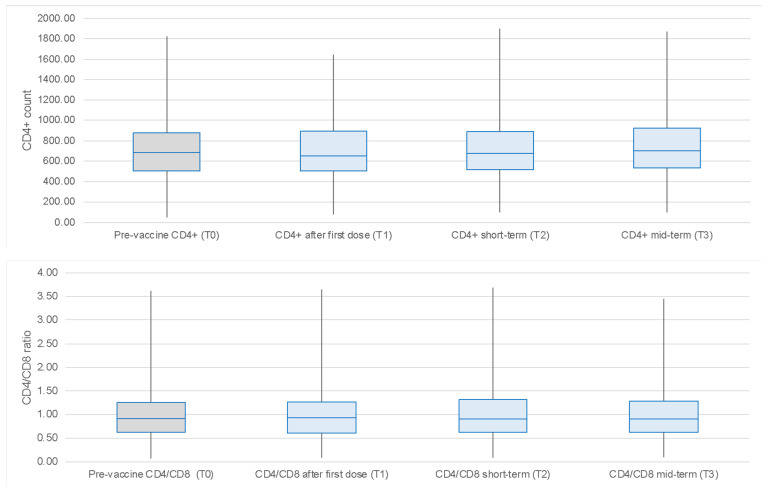
CD4+ count (**top**) and CD4/CD8 ratio (**bottom**) at each follow-up time point (T0 to T3). The boxplots illustrate the distribution of both the CD4+ count and CD4/CD8 ratio across the different time points. For the CD4+ count, at pre-vaccination (T0), the median is 687.0, with an interquartile range (IQR) from 508.0 to 875.0. After the first dose (T1), the median is 654.0, with an IQR from 508.0 to 891.5. At short-term follow-up (T2), the median is 676.0, with an IQR from 518.0 to 888.5. Finally, at mid-term (T3), the median is 703.0, with an IQR from 536.0 to 921.0. For the CD4/CD8 ratio, at pre-vaccination (T0), the median is 0.91, with an IQR from 0.62 to 1.25. After the first dose (T1), the median is 0.93, with an IQR from 0.61 to 1.27. At short-term (T2), the median is 0.90, with an IQR from 0.62 to 1.32. At mid-term (T3), the median is 0.90, with an IQR from 0.62 to 1.28.

The CD4 count and CD4/CD8 ratio at each time point during the follow-up period are shown in Figure 3 (upper and lower sections, respectively). The CD4 cell count and CD4/CD8 ratio recorded at T0 (i.e., before vaccination) were used as reference values for statistical analysis. Linear regression analysis revealed no statistically significant relationship between anti-S IgG levels and CD4 count or CD4/CD8 ratio either in the short or long term at each time point examined (T1–T4) (*p*-values short term: 0.52, 0.57, and 0.99, respectively; medium term: 0.71, 0.86, and 0.99).

### 3.5. Predictors of Anti-S IgG Concentration

The probability of a lower anti-S antibody response appears to be related to the presence of dyslipidemia at T2 (aOR 4.75, 95% CI: 1.39–16.20), as well as diabetes (aOR 7.11, 95% CI: 1.10–46.1) at T3 (Supplementary Table S2). Conversely, a 1-point increase in BMI (aOR 0.78; 95% CI: 0.64–0.95), undergoing therapy with PI inhibitors (AORs 0.02, 95% CI: 0.002–0.32), and being female (aOR 0.06, 95% CI: 0.003–0.96) were protective factors against a low antibody response to vaccination at T3. Finally, at the long-term T4 time point, no variables were statistically significant in determining the risk or level of protection against obtaining a reduced antibody response (Supplementary Table S2).

Figure 2a,b illustrate the antibody concentrations measured in all 165 participants, expressed in Arbitrary Units per milliliter (AU/mL). In Figure 2a, data are represented through boxplots which illustrate the median, the interquartile range (IQR), and the extremes for each time point: one month (T1), two months (T2), four months (T3), and seven months (T4) after the first dose of vaccination. Each boxplot illustrates variations in antibody levels over time. The variations in antibody levels over time are highlighted, with specific coloring at T4: green for participants without a booster, showing a more contained range, and orange for those with a booster, indicating a significant median increase. In Figure 2b, the same data are presented using a trajectory plot. This representation allows for the visualization of individual variations in antibody concentrations across all participants, making it easier to observe the distinct patterns between those who received the booster and those who did not.

This figure shows the CD4+ T cell counts and CD4/CD8 ratios across the designated time points (T0 to T3) for the study cohort. Median values and interquartile ranges (IQRs) for both CD4+ counts and CD4/CD8 ratios remain relatively stable, with no significant fluctuations observed during the study period, including for the time points immediately following vaccination doses.

## 4. Discussion

The immune response to vaccination in PLWH is of paramount importance for optimizing their health care and improving outcomes. Vaccination is essential in preventing infectious diseases, but the efficacy and immune response in PLWH may vary compared to the general population.

Research, including several studies and a meta-analysis, has shown the efficacy of the BNT162b2 vaccine in eliciting a robust immune response in PLWH, with antibody levels typically exceeding the 50 AU/mL threshold in all subjects after completing the vaccination cycle [38,44,45,46].

Consistent with these findings, our research demonstrates that all participants in the study exhibited a robust response to vaccination, exceeding the designated threshold value upon completion of the primary cycle. Remarkably, even the four individuals who did not exhibit a response after the first dose displayed positive antibody levels subsequent to receiving a second dose within the primary cycle. This highlights the efficacy and potential benefit of a two-dose regimen in eliciting a protective immune response in individuals who may initially show limited responsiveness to the initial vaccine dose, potentially lowering the risk of severe COVID-19 outcomes.

In addition, even though our study did not measure either neutralization activity or the protective effect against SARS-CoV-2 infection and/or severe disease, antibody levels exponentially higher than the positive cut-off for anti-S IgG (50 AU/mL) may have a role in terms of potential neutralizing activity and ensuring protection from severe COVID-19. Different antibody thresholds may be necessary for direct protection against infection or transmission. Further research is warranted in both scenarios to explore this further.

Regarding immunological parameters, the CD4+ cell count and CD4/CD8 ratio remained stable during the study, confirming the safety of the vaccine in this immunocompromised population. Indeed, no other serious side effects were reported during the study period.

Another point worth noting is the durability of the immune response following vaccination. A study that analyzed the long-term antibody response to the BNT162b2 vaccine in immunocompromised COVID-19-naïve subjects, including PLWH [47], found that the vaccine was effective in inducing a strong and sustained antibody response in HIV patients. However, it was observed that antibody titers tend to decline over time (approximately 4–6 months), which underscores the importance of administering booster doses to maintain lasting immunity [48,49]. Consistent with these findings, our study shows divergent trends in antibody levels between subjects who received a booster dose within 180 days of the primary vaccination cycle and those who received no additional dose, with the former showing significantly higher antibody levels. These data underline the importance of the timely administration of booster doses to maintain higher antibody concentrations, as already supported by the current scientific literature [50]. Unfortunately, we did not compare the antibody response between our cohort of PLWH and the general population. However, humoral responses in the general population following COVID-19 vaccination decreased in all age groups after six months, and a continuous waning in humoral responses was estimated primarily in the older population and in individuals with a delayed administration of the second vaccine dose [51].

In relation to neutralizing activity, while our study did not directly measure neutralization, it is notable that anti-Spike IgG concentrations exponentially higher than the positivity cut-off of 50 AU/mL were observed in a significant proportion of participants. As shown in Figure 2, many participants reached this threshold, particularly after the second dose at the T2 time point, completing the primary vaccination cycle, and at T4 for those who received a booster dose. These findings are consistent with the expected serological responses following vaccination and suggest an increased neutralizing capacity, which may be indicative of enhanced protective immunity among the participants.

Regarding sociodemographic, pathological, and treatment-related factors, our regression analysis identified several variables influencing antibody responses. Specifically, patients with dyslipidemia were more likely to be in the lower quartile of humoral response in the short term, while diabetes was associated with a reduced antibody response in the medium term. These findings align with recent scientific evidence indicating that the existence of comorbidities in immunocompromised patients correlates with an initial weaker response [36,52]; moreover, dyslipidemia and diabetes are known to be associated with immune dysregulation, immune inflammation, and immunosuppression [53,54]. Conversely, the use of HIV protease inhibitors, a higher BMI, and being female were associated with a lower likelihood of being in the bottom quartile of antibody response.

Several studies during the COVID-19 pandemic have explored repurposing HIV protease inhibitors against SARS-CoV-2, showing a certain in vitro activity but lacking clinical efficacy [55,56,57,58,59]. There is no established evidence linking these therapies to improved vaccination responses. Higher antibody titers in PI-treated PLWH may indicate the need for further investigations into the impact of different cART regimens on immune status, including both vaccine-induced cell-mediated and humoral immunity. Instead, a higher BMI in PLWH may be linked to enhanced vaccination responses due to better nutritional status and the reduced risk of immunocompromising conditions like cachexia [60].

These findings underscore the crucial need to tailor vaccination strategies, particularly for people living with HIV, to enhance their immune response and attain sustained antibody protection over time [10,45]. As proposed by Tortellini et al. [61], the use of booster doses may be an effective approach that could help overcome potential waning immunity and enhance long-term protection against infectious diseases. However, determining the optimal timing and frequency of booster doses requires further research.

This study has some limitations. The sample size is relatively small, consisting only of PLWH who were being treated at a single medical facility and primarily involving male participants, which limits the generalizability of the findings. In addition, the study is focused on a homogeneous group of PLWH treated with effective antiretroviral therapy and with no individuals having a CD4+ cell count < 200, precluding the assessment of how different immunosuppression levels might influence vaccine response.

Despite these limitations, we are confident that this particular group offers a unique opportunity to study distinctive traits and sets the groundwork for further research into the mechanisms underlying the observed associations between vaccination and antibody-mediated immune response.

## 5. Conclusions

In summary, the study results show that people living with HIV (PLWH) exhibited a strong antibody response to the BNT162b2 mRNA COVID-19 vaccine. All participants maintained antibody levels above the threshold (>50 AU/mL) during the six-month follow-up period, which further increased after receiving the booster dose. These findings endorse the inclusion of this vulnerable population in vaccination programs, providing confidence in the ability of vaccinations to generate an effective immune response.

## Figures and Tables

**Table 1:** Baseline characteristics and comorbidities of the study population.

Sample Characteristics	*n* = 165	%	Median	P25	P75
Age			55.0	47.0	62.0
Male	137	83.0%			
Italian	150	90.9%			
BMI			25.2	22.9	27.8
Years lived with HIV			16.0	8.0	24.0
Initiated cART within one year of diagnosis	130	78.8%			
No AIDS event	121	74.2%			
Nadir CD4+ T cell count			270.0	140.0	421.0
CD4 > 500 cells/mm^3^	127	77.0%			
CD4 351–500 cells/mm^3^	23	13.9%			
CD4 200–350 cell/mm^3^	15	9.1%			
CD4+ T-cell count (T0)			687.0	508.0	875.0
CD4/CD8 ratio (>0.5)	135	81.8%			
CD4/CD8 (T0)			0.9	0.6	1.3
HIV-RNA at T0 (copies/mm^3^)			0.0	0.0	20.0
HIV-RNA (<50 copies/mm^3^)	161	97.6%			
INI (integrase inhibitors)-based ART	94	57.0%			
PI (protease inhibitors)-based ART	33	20.0%			
NNRTI (non-nucleoside reverse transcriptase inhibitor)-based ART	60	36.4%			
Dual therapy	49	29.7%			
Triple therapy	110	66.7%			
Dyslipidemia	54	32.7%			
Cardiovascular disease	60	36.4%			
Depression	11	6.7%			
Hepatopathy	25	15.2%			
Pneumological diseases	4	2.4%			
Renal failure/dialysis	3	1.8%			
Diabetes	22	13.3%			
Solid tumours	28	17.0%			
Hematological neoplasms	4	2.4%			
Autoimmunity	13	7.9%			
Hepatitis C virus antibodies (positive)	37	22.4%			
Antibodies to hepatitis B core antigen (positive)	65	40.6%			
Hepatitis B surface antigen (positive)	3	1.9%			
Treponema pallidum antibodies (positive)	59	36.6%			
Venereal Disease Research Laboratory Test (positive)	10	6.3%			

## Data Availability

All data are available from the corresponding author upon reasonable request.

## Supplementary Materials

The following supporting information can be downloaded at https://www.mdpi.com/article/10.3390/vaccines12101172/s1, Table S1: Baseline characteristics and comorbidities of the study population. Table S2: Logistic regression analysis of factors affecting anti-Spike IgG concentrations.
