# Supplementary material for: Immunogenicity and Determinants of Antibody Response to the BNT162b2 mRNA Vaccine: A Longitudinal Study in a Cohort of People Living with HIV
Source: Vaccines (Basel). 2024 Oct 16;12(10):1172. doi: 10.3390/vaccines12101172 (PMC11512344; doi:10.3390/vaccines12101172)
Supplement: Supplementary file 1 [file vaccines-12-01172-s001.zip › vaccines-3171699-supplementary.pdf]

vaccines

Supplementary Materials

Table S1. Baseline characteristics and comorbidities of the study population. Table presents a detailed comparison of baseline characteristics and comorbidities between the boosted and non-boosted groups. It includes the means and standard deviations for continuous variables and percentages for categorical variables. Key sociodemographic factors (age, sex, nationality, years living with HIV), clinical parameters (CD4+ T cell count, CD4/CD8 ratio, HIV-RNA levels), and comorbidities (cardiovascular disease, dyslipidemia, diabetes, renal failure, solid/hematologic tumors) are displayed. Statistically significant differences are highlighted with p-values below 0.05 and 0.01. This table complements the simplified version in the manuscript, providing a comprehensive overview of both the overall study population and the subgroups (boosted and non-boosted).

		Total				No boost (n = 143)				Boost (n = 22)			
		**Mean**	**SD**	**n**	**%**	**Mean**	**SD**	**n**	**%**	**Mean**	**SD**	**n**	**%**
Age		54.0	11.2			53.9	10.8			54.4	13.4		
Sex (0: M; 1: F)	0			137	83.0%			115	80.4%			22	100.0% **
	1			28	17.0%			28	19.6%			0	0.0%
Nationality (0: Italian; 1: foreign)	0			150	90.9%			130	90.9%			20	90.9%
	1			15	9.1%			13	9.1%			2	9.1%
BMI		25.8	4.1			25.8	4.2			25.8	3.8		
Years lived with HIV		16.9	9.8			16.7	9.6			17.9	11.4		
Initiated cART within one year of diagnosis	0			130	78.8%			115	80.4%			15	68.2%
	1			35	21.2%			28	19.6%			7	31.8%
AIDS Event (0: Yes, 1: No)	0			121	74.2%			107	75.9%			14	63.6%
	1			42	25.8%			34	24.1%			8	36.4%
Nadir CD4+ T cell count		309.6	229.7			316.0	225.5			268.7	257.0		
CD4 > 500 cells/mm3 (0: > 500, 1: < 500)	0			127	77.0%			111	77.6%			16	72.7%
	1			38	23.0%			32	22.4%			6	27.3%
CD4+ T-cell count (T0)		720.6	298.0			724.8	299.9			693.4	290.5		
CD4/CD8 ratio (> 0.5) (0: > 0.5, 1: < 0.5)	0			135	81.8%			118	82.5%			17	77.3%
	1			30	18.2%			25	17.5%			5	22.7%
CD4/CD8 (T0)		1.02	0.62			1.05	0.65			0.81	0.36		

HIV-RNA (<50 copies/mm3) (0: < 50, 1: > 50)	0			161	97.6%			139	97.2%			22	100.0%
	1			4	2.4%			4	2.8%			0	0.0%
HIV-RNA at T0 (copies/mm3)		10.3	23.1			9.9	24.2			12.7	14.6		
INI (Integrase Inhibitors)-based ART (0: No, 1: Yes)	0			71	43.0%			61	42.7%			10	45.5%
	1			94	57.0%			82	57.3%			12	54.5%
PI (Protease Inhibitors)-based ART (0: No, 1: Yes)	0			132	80.0%			113	79.0%			19	86.4%
	1			33	20.0%			30	21.0%			3	13.6%
NNRTI (Non-Nucleoside Reverse Transcriptase Inhibitors)-based ART (0: No, 1: Yes)	0			105	63.6%			91	63.6%			14	63.6%
	1			60	36.4%			52	36.4%			8	36.4%
Dual Therapy (0: No, 1: Yes)	0			116	70.3%			100	69.9%			16	72.7%
	1			49	29.7%			43	30.1%			6	27.3%
Triple Therapy (0: No, 1: Yes)	0			55	33.3%			48	33.6%			7	31.8%
	1			110	66.7%			95	66.4%			15	68.2%
Dyslipidemia (0: No, 1: Yes)	0			111	67.3%			93	65.0%			18	81.8%
	1			54	32.7%			50	35.0%			4	18.2%
Cardiovascular Disease	0			105	63.6%			92	64.3%			13	59.1%

(0: No, 1: Yes)	1	60	36.4%	51	35.7%	9	40.9%
Depression (0: No, 1: Yes)	0	154	93.3%	133	93.0%	21	95.5%
	1	11	6.7%	10	7.0%	1	4.5%
Hepatopathy (0: No, 1: Yes)	0	140	84.8%	123	86.0%	17	77.3%
	1	25	15.2%	20	14.0%	5	22.7%
Pneumological Diseases (0: No, 1: Yes)	0	161	97.6%	140	97.9%	21	95.5%
	1	4	2.4%	3	2.1%	1	4.5%
Renal Failure / Dialysis (0: No, 1: Yes)	0	162	98.2%	140	97.9%	22	100.0%
	1	3	1.8%	3	2.1%	0	0.0%
Diabetes (0: No, 1: Yes)	0	143	86.7%	126	88.1%	17	77.3%
	1	22	13.3%	17	11.9%	5	22.7%
Solid Tumours (0: No, 1: Yes)	0	137	83.0%	118	82.5%	19	86.4%
	1	28	17.0%	25	17.5%	3	13.6%
Haematological Neoplasms (0: No, 1: Yes)	0	161	97.6%	141	98.6%	20	90.9%
	1	4	2.4%	2	1.4%	2	9.1%
Autoimmunity (0: No, 1: Yes)	0	152	92.1%	132	92.3%	20	90.9%
	1	13	7.9%	11	7.7%	2	9.1%
Hepatitis C Virus antibody (0: No, 1: Yes)	0	128	77.6%	111	77.6%	17	77.3%
	1	37	22.4%	32	22.4%	5	22.7%
Antibody to Hepatitis B Core Antigen	0	95	59.4%	81	58.3%	14	66.7%

(anti-HBc positive) (0: No, 1: Yes)	1	65	40.6%	58	41.7%	7	33.3%
Active Hepatitis B Infection Surface Antigen (HBsAg) (0: No, 1: Yes)	0	158	98.1%	138	98.6%	20	95.2%
	1	3	1.9%	2	1.4%	1	4.8%
Treponema pallidum Hemaggluti nation Assay (TPHA positivity) (0: No, 1: Yes)	0	102	63.4%	92	65.7%	10	47.6%
	1	59	36.6%	48	34.3%	11	52.4%
Venereal Disease Research Laboratory (VDRL positivity) (0: No, 1: Yes)	0	150	93.8%	132	95.0%	18	85.7%
	1	10	6.3%	7	5.0%	3	14.3%

* p-value < 0.1
** p-value < 0.05

Table S2. Logistic regression analysis of factors affecting anti-spike IgG concentrations. Table presents the results of the logistic regression analysis performed at each time point (T2, T3, T4) to evaluate factors associated with the probability of being in the lowest quartile for anti-Spike IgG concentrations. . The dependent variable is the risk of low antibody response, with independent variables including demographic factors (sex, BMI), ART regimens (e.g., INI, PI, NNRTI), and comorbidities (e.g., dyslipidemia, cardiovascular disease, diabetes, liver disease, renal insufficiency, solid/hematologic tumors). Also viro-immunological parameters were included in the model. Both unadjusted and adjusted odds ratios (OR and aOR) with 95% confidence intervals (CI) are provided. Protective factors, where the confidence interval is entirely below 1, are highlighted in green. Risk factors, where the confidence interval is entirely above 1, are shown in red.

	T2 (short term)						T3 (mid term)						T4 (long-term, no boost group only)					
	Unadjusted OR	95% C.I.for EXP(B)		AOR	95% C.I. for EXP(B)		Unadjusted OR	95% C.I.for EXP(B)		AOR	95% C.I. for EXP(B)		Unadjusted OR	95% C.I. for EXP(B)		AOR	95% C.I. for EXP(B)	
**Sex**	0.187	0.042	0.823	0.152	0.012	2.018	0.100	0.013	0.763	**0.057**	0.003	0.955	0.432	0.139	1.344	0.188	0.018	2.013
**BMI**	0.940	0.859	1.029	0.857	0.726	1.011	0.951	0.867	1.044	**0.780**	0.643	0.946				0.918	0.751	1.121
INI (Integrase Inhibitors)-based ART	0.887	0.438	1.795	1.328	0.208	8.497	1.050	0.504	2.187	0.207	0.022	1.937	0.578	0.270	1.237	0.127	0.008	2.096
**PI (Protease Inhibitors)-based ART**	0.922	0.380	2.238	0.650	0.095	4.446	0.536	0.191	1.500	**0.025**	0.002	0.322	0.529	0.186	1.505	0.090	0.006	1.369
NNRTI (Non-Nucleoside Reverse Transcriptase Inhibitors)-based ART	1.446	0.707	2.959	1.504	0.296	7.644	0.885	0.414	1.894	0.155	0.020	1.225	1.841	0.853	3.971	0.252	0.021	3.047
Dual Therapy	1.083	0.506	2.318	0.206	0.014	3.078	0.675	0.292	1.558	0.053	0.001	2.422	1.014	0.455	2.258	1.275	0.007	247.084

Triple Therapy	0.657	0.318	1.358	0.169	0.011	2.600	1.301	0.590	2.869	0.146	0.003	6.576	1.014	0.455	2.258	0.813	0.005	140.288
**Dyslipidemia**	2.380	1.155	4.904	**4.754**	1.395	16.198	1.472	0.694	3.121	1.845	0.478	7.112	1.069	0.486	2.352	2.222	0.483	10.228
Cardiovascular Disease	1.265	0.616	2.597	0.516	0.134	1.986	1.581	0.756	3.306	1.074	0.261	4.417	1.409	0.649	3.056	0.906	0.175	4.691
Depression	1.106	0.279	4.376	2.168	0.295	15.934	1.275	0.321	5.065	1.717	0.182	16.214	2.104	0.559	7.925	0.314	0.005	18.203
Hepatopathy	0.696	0.244	1.988	0.293	0.044	1.945	1.066	0.392	2.894	6.111	0.753	49.607	1.745	0.636	4.786	5.942	0.334	105.584
Pneumological Diseases	3.025	0.413	22.180	6.670	0.410	108.519	3.472	0.472	25.521	12.386	0.556	276.105	6.235	0.548	70.921	10.888	0.354	335.091
**Diabetes**	2.308	0.906	5.876	5.023	0.941	26.797	2.721	1.060	6.985	**7.114**	1.098	46.099	1.745	0.595	5.119	3.258	0.447	23.728
Solid Tumours	0.971	0.380	2.481	1.069	0.197	5.789	0.895	0.334	2.398	1.438	0.227	9.098	0.926	0.338	2.536	3.499	0.578	21.183
Autoimmunity	1.333	0.388	4.578	1.753	0.255	12.049	0.586	0.124	2.767	0.207	0.020	2.100	0.277	0.034	2.244	0.306	0.020	4.742
Hepatitis C Virus antibody	0.620	0.250	1.540	0.784	0.173	3.547	0.730	0.292	1.826	0.739	0.139	3.937	1.217	0.503	2.947	1.797	0.252	12.825
Antibody to Hepatitis B Core Antigen (anti-HBc positive)	1.133	0.555	2.313	1.378	0.366	5.189	1.248	0.598	2.604	1.505	0.387	5.862	1.243	0.575	2.690	0.761	0.166	3.483
Treponema pallidum Hemagglutinati	1.865	0.911	3.817	2.203	0.587	8.264	1.800	0.860	3.769	1.388	0.358	5.377	1.636	0.746	3.591	1.853	0.326	10.543

on Assay (TPHA positivity)																		
Venereal Disease Research Laboratory (VDRL positivity)	2.036	0.545	7.610	6.055	0.875	41.918	1.408	0.346	5.736	1.666	0.211	13.129	4.344	0.922	20.469	5.254	0.450	61.351
AIDS Event	0.792	0.341	1.839	0.624	0.143	2.719	0.746	0.311	1.792	0.321	0.057	1.824	0.912	0.369	2.253	0.691	0.096	4.956
Nadir CD4+ T cell count	1.001	0.999	1.002	1.002	1.000	1.005	1.000	0.999	1.002	1.001	0.998	1.004	1.001	0.999	1.002	1.002	0.999	1.006
CD4 < 500 cells/mm^3	0.884	0.379	2.061	1.103	0.282	4.320	1.263	0.548	2.911	0.898	0.185	4.364	1.217	0.503	2.947	1.820	0.308	10.774
CD4/CD8 ratio < 0.5	1.325	0.553	3.174	2.056	0.567	7.457	1.273	0.515	3.147	0.751	0.183	3.079	1.513	0.590	3.877	0.597	0.108	3.287
HIV-RNA >50 copies/mm^3	3.025	0.413	22.180	9.545	0.712	127.924	3.472	0.472	25.521	1.803	0.123	26.482	3.088	0.419	22.768	7.990	0.403	158.449
